# Brevican “nets” voltage-gated calcium channels at the hair cell ribbon synapse

**DOI:** 10.1186/s12915-018-0575-7

**Published:** 2018-09-26

**Authors:** Thomas M. Coate, Katherine Conant

**Affiliations:** 1 Department of Biology, Georgetown University, 37th and O St. NW, Washington, DC, 20007 USA; 2 Department of Neuroscience, Georgetown University Medical Center, 3970 Reservoir Road, Washington, DC, 20007 USA

## Abstract

During hearing in mammals, “sensorineural” inner hair cells convert sound wave-generated mechanical input into electrical activity, resulting in glutamate release onto type I spiral ganglion neurons (SGNs) at specialized synapses known as “ribbon synapses”. New findings published here in *BMC Biology* by Sonntag and colleagues indicate a role for the proteoglycan Brevican in forming perineurounal net (PNN) baskets at these synapses and controlling the spatial distribution of presynaptic voltage-gated calcium channels that regulate glutamate release. These findings may provide insight into the mechanism by which individual ribbon synapses within a single hair cell can function in an independent manner to facilitate hearing within a broad dynamic range.

## Commentary

**Fig. 1:**
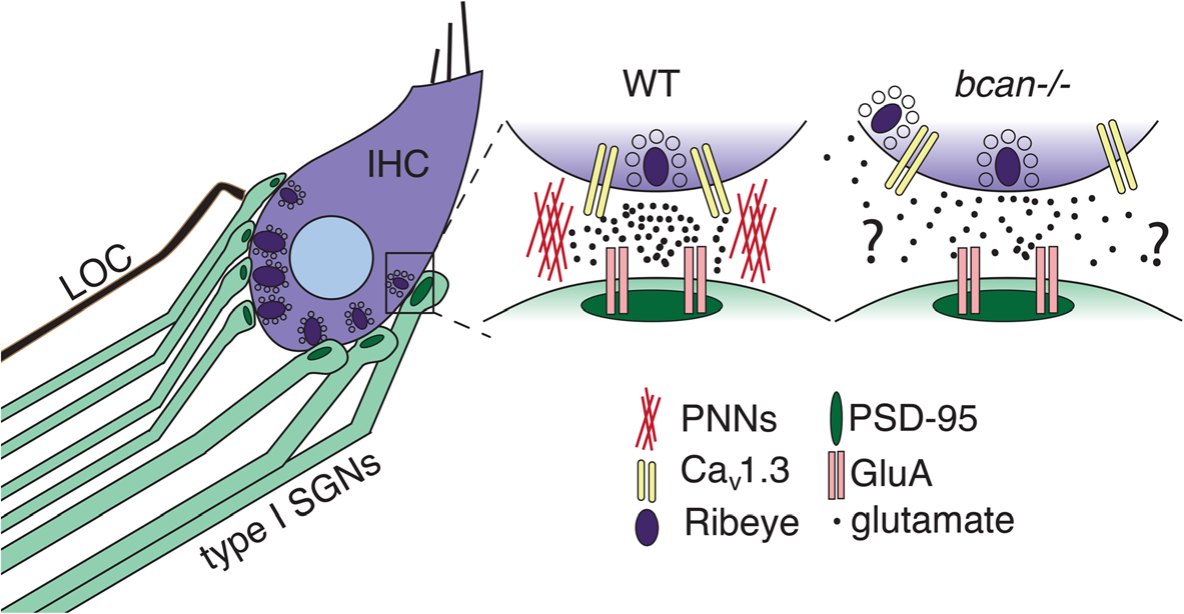
Overview of the IHC-SGN synapse. Shown is a cochlear inner hair cell with SGN contacts. In the expanded view of a wild-type (*WT*) contact, an individual release site of a ribbon synapse is shown with alignment of presynaptic Ca_v_1.3 channels and the post-synaptic density of an SGN contact. This arrangement likely facilitates spatially precise neurotransmitter release. With injury or other conditions that may increase expression of PNN-targeting extracellular proteases, Cav1.3 channels could be misaligned and spatially precise transmitter release impaired in a manner similar to that shown in the expanded view of a *bcan*−/− contact. A lateral olivocochlear (LOC) efferent contact to an SGN dendrite is also shown

The base of an adult mouse inner hair cell (IHC) contains around 20 presynaptic ribbon bodies, each of which is dominated by an electron-dense spherical structure covered in glutamate-containing synaptic vesicles (Fig. 1). Along the landscape of the inner hair cell, the ribbon bodies and postsynaptic densities that juxtapose them show remarkable heterogeneity in size, and differences in size are known to correlate with their spatially distinct positions within the inner hair cell [1]. Type I SGNs, the primary afferents of the auditory system, are also heterogeneous and can be broken into three classes based on a variety of features, including physiological parameters, morphology, and molecular profiles [2]. The ribbon presynaptic region contains a plethora of scaffolding and regulatory factors necessary for glutamate release, including Bassoon, Ribeye, Vglut3, Ca_v_1.3, and many others. The molecular composition of the postsynaptic density includes scaffolding proteins like Shank1 and PSD95, and although nearly all forms of glutamate receptors are expressed by the SGNs, AMPA-type glutamate receptors are almost entirely responsible for their excitability. The beautiful immunostaining studies shown by Sonntag et al. [3] show that PNN proteins such as Brevican, Aggrecan, and HAPLN1 are present around the hair cell ribbon synapse where they may prevent glutamate spillover to neighboring synapses. These proteins have not been well characterized in the peripheral auditory system, and the data in this report suggest they may be important regulators of auditory afferent transmission.

PNNs are a specialized form of dense extracellular matrix (ECM) that surround select neuronal subpopulations within the central nervous system (CNS). These lattice-like structures predominantly associate with neuronal types that are fast spiking and metabolically active, and thus they have been well studied for their ability to protect enveloped cells from oxidant stress [4]. Recent work also suggests that, since PNNs closely border synaptic contacts, their presence can facilitate the reliability and temporal precision of fast synaptic transmission. Consistent with this, PNN disruption has been shown to enhance the lateral mobility of GluAs, which may reduce post-synaptic receptor engagement [5]. It has also been suggested PNN disruption can enhance synaptically released glutamate dispersion Indeed, in mice deficient for the PNN component Brevican, excitatory input from pyramidal cells to PNN-surrounded parvalbumin-expressing fast spiking interneurons is reduced [6].

Sonntag et al. now report the presence of basket-like ECM structures that contain classic PNN components, including Brevican external to the CNS and specifically at the synaptic poles of inner hair cells (IHCs). Given that IHC signaling is high frequency and continuous [7], this localization lends additional support to the idea that PNNs support the fidelity of synaptic transmission at sites that require faithful transmission and high temporal precision. Ca_v_1.3 proteins tightly cluster around individual ribbon synapses and are necessary for proper glutamate release at those discrete sites [8]. Sonntag et al. show that, in Brevican-deficient mice, the colocalization of presynaptic Ca_v_1.3 and post-synaptic density-95 labeling is disrupted, and presumably this dislocation leads to defects in afferent firing. If this were the case, it would be reasonable to conclude that PNN integrity is needed to support the spatial fidelity of glutamatergic neurotransmission.

We note here that cochlear efferents (arising from the lateral superior olivary complex) also form synapses on the endings of the type I SGNs, adjacent to the afferent synapses. The lateral efferents are known to release several neurotransmitters, including acetylcholine and dopamine, that may be important protectors against auditory neuropathy [9] and are in very close proximity to the apparent location of the PNN proteins. While PNNs have been best studied for their effects on glutamatergic transmission [5], it is possible that the PNNs introduced in this report also interact with and influence the lateral efferent synapses in some manner.

The IHC ribbon synapse is relatively unique. In particular, neurotransmitter release sites exist and are compartmentalized within a single cell such that each afferent is activated by only the presynaptic ribbon adjacent to it. This differs from other types of hair cells, like type I hair cells in the vestibular system, which are innervated by an afferent calyx that receives glutamate released from multiple ribbons. Furthermore, the firing properties of each afferent are highly variable, and pairs of afferents contacting the same inner hair cell do not show any correlations in activity [10]. Thus, each ribbon body–afferent pairing can be assumed to act as an individual channel within a single inner hair cell. Sonntag et al. propose that unique Brevican baskets may separate these individual sites and potentially prevent glutamate spillover to preserve appropriate transmission within a large dynamic range.

The finding that PNNs may affect transmission at the IHC-SGN synapse has several implications. For example, in ototoxic noise exposure an increase in cochlear reactive oxygen and nitrogen species is observed. These species may in turn upregulate the expression of PNN-degrading proteases to influence synaptic transmission and/or cell viability.

At least three important questions in the area of PNNs and auditory afferent transduction should now be addressed. First, how and when do PNNs assemble in the context of ribbon synapse formation in the cochlea? In mouse, initial connectivity between SGNs and hair cells occurs starting around E14.5, and the maturation of the ribbon synapses occurs from around P0 through P10 (when hearing commences). During this latter phase, the SGNs engage in an elaborate series of events including branching refinement, spontaneous firing, myelination by Schwann cells, synaptic pruning, and innervation by efferents. After hearing onset, afferent firing characteristics and the ribbon synaptic structures go through a protracted phase of maturation through around P30 [1, 10]. In the future, it will be important to determine the timeline of PNN formation in the context of auditory development and the extent to which any of these events affect PNNs or vice versa. This could enhance our understanding of the cellular and molecular role(s) of PNNs in ribbon synapse formation and may help determine if PNNs could be useful therapeutic targets in efforts to re-wire the cochlea following damage. Second, it will be of interest to evaluate how loss of the perineuronal baskets affects afferent firing. In this paper, the authors showed convincingly there were no changes in inner hair cell currents in the absence of *bcan* (Brevican). But, perhaps more important—and considerably more difficult experimentally—would be to examine afferent firing features such as AMPA-dependent excitatory postsynaptic currents (EPSCs) in the absence of *bcan*. Sonntag et al. did not show severely elevated auditory brainstem response (ABR) thresholds in the *bcan* knockout mice, but tests of ABR threshold are generally low resolution and thus might not reveal subtle changes in afferent excitability. Third, PNNs and the *bcan* knockout model should also be examined in the context of auditory neuropathy stemming from noise induced- or age-related hearing loss.

In summary, Sonntag et al. have demonstrated a new player in IHC to SGN transmission and one that warrants future study to better understand its role in normal physiology and pathology.
